# Immunotherapy Associated Neurotoxicity in Pediatric Oncology

**DOI:** 10.3389/fonc.2022.836452

**Published:** 2022-02-21

**Authors:** Haneen Shalabi, Anandani Nellan, Nirali N. Shah, Juliane Gust

**Affiliations:** ^1^ National Cancer Institute, Pediatric Oncology Branch, National Institutes of Health, Bethesda, MD, United States; ^2^ Seattle Children’s Research Institute, Seattle, WA, United States; ^3^ Department of Neurology, University of Washington, Seattle, WA, United States

**Keywords:** immunotherapy, pediatric cancer, adoptive cell therapy, antibody therapy, neurotoxicity, vaccine therapy

## Abstract

Novel immunotherapies are increasingly being employed in pediatric oncology, both in the upfront and relapsed/refractory settings. Through various mechanisms of action, engagement and activation of the immune system can cause both generalized and disease site-specific inflammation, leading to immune-related adverse events (irAEs). One of the most worrisome irAEs is that of neurotoxicity. This can present as a large spectrum of neurological toxicities, including confusion, aphasia, neuropathies, seizures, and/or death, with variable onset and severity. Earlier identification and treatment, generally with corticosteroids, remains the mainstay of neurotoxicity management to optimize patient outcomes. The pathophysiology of neurotoxicity varies across the different therapeutic strategies and remains to be elucidated in most cases. Furthermore, little is known about long-term neurologic sequelae. This review will focus on neurotoxicity seen with the most common immunotherapies used in pediatric oncology, including CAR T cell therapy, alternative forms of adoptive cell therapy, antibody therapies, immune checkpoint inhibitors, and tumor vaccines. Herein we will discuss the incidence, pathophysiology, symptomatology, diagnosis, and management strategies currently being utilized for immunotherapy-associated neurotoxicity with a focus on pediatric specific considerations.

## Introduction

**Figure 1:**
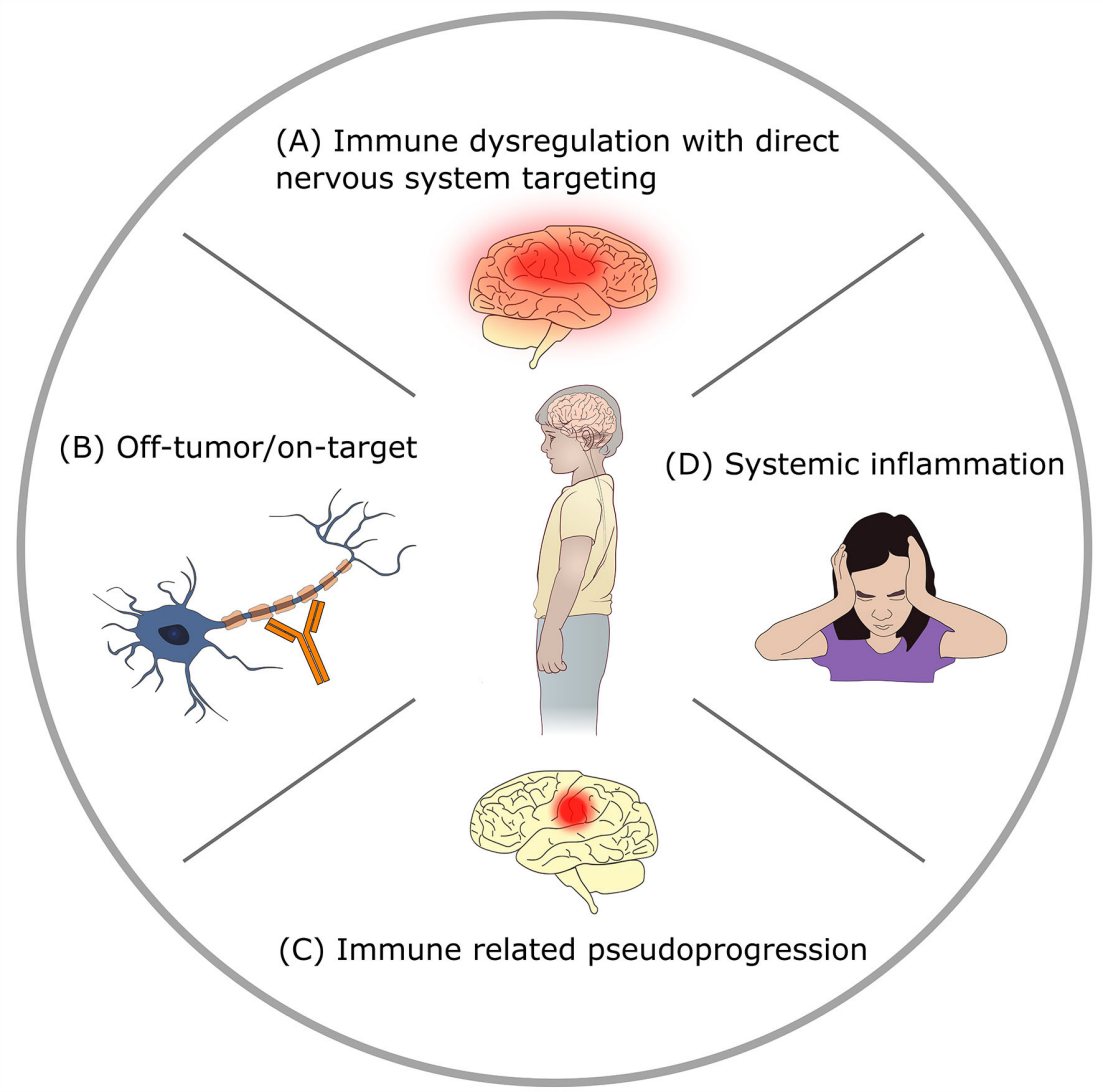
Mechanisms of neurotoxicity in immunotherapy. **(A)** Direct targeting of the nervous system by immune dysregulation: immunotherapy provokes autoimmunity or other immune-mediated CNS injury. Examples: checkpoint-blockade related encephalitis, immune effector cell associated neurotoxicity syndrome (ICANS). **(B)** Off-tumor on-target toxicity: immunotherapy agent targets normal tissue. Example: GD2 antibody mediated peripheral neuropathy. **(C)** Immune related pseudoprogression: targeting of tumor by immunotherapy causes peritumoral inflammation with increased edema. Example: tumor inflammation associated neurotoxicity (TIAN) related to GD2-CAR T therapy for brain tumors. **(D)** Systemic inflammation related neurologic signs and symptoms: neurologic symptoms in the setting of elevated systemic inflammatory states with absence of direct CNS injury. Example: headache after anti-tumor vaccination.

The rapid emergence of novel immunotherapies has dramatically shifted the treatment paradigm in oncology, particularly in those with relapsed/refractory disease where standard therapies have failed. These novel therapies strive to overcome chemotherapeutic and radiotherapy resistance by harnessing the immune system to treat malignancies. Approaches include direct engagement of the immune system, indirect activation of the intrinsic immune response, or targeted delivery of anti-cancer therapeutics. We also include select non-immunotherapy based targeted antibody therapies in this review, such as bevacizumab, alongside other immunotherapeutic agents, given the side effect profiles and indications for use significantly overlaps with that of immunotherapies. With novel mechanisms of action, a host of unique adverse events have also materialized. Generally categorized as immune-related adverse events (irAEs), the constellation of toxicities is broad and includes inflammation-mediated manifestations, components of autoimmunity, and/or on-target/off-tumor effects amongst other etiologies (**Table 1**) (1, 2). Presenting as generalized and/or disease site-specific toxicities, irAEs may include localized inflammation at the disease site or multi-organ system involvement (2–4). Neurotoxicities are amongst the irAEs which are often the most worrisome and least understood (5–10). Neurologic irAEs can be grouped into 4 mechanistic categories (**Figure 1**): direct targeting of the nervous system due to immune dysregulation, off-tumor on-target toxicity, immune related pseudoprogression, or neurologic symptoms attributable to systemic inflammatory states. The exact mechanism of specific toxicities is often not completely clear, and there may be combination and overlap syndromes.

**Table 1:** Types of immunotherapy, mechanism of action, systemic toxicity.

**Adoptive cell therapy**
*Category Type and Mechanism of Action*
CAR T cell therapy: Synthetic receptors that combine antibody recognition properties of B-cell and effector functions of a T-cell. The single chain variable fragment (scFv) is directed against specific cell surface antigens to which it binds, leading to and T-cell activation, expansion, and target cell elimination occurs.
T cell receptor therapy: Genetically engineered T-cell receptors that can recognize specific antigens, either intracellularly or extracellularly. They are MHC restricted as they depend on presentation by MHC molecules to recognize targets and activate T-cell function.
*Toxicity Profile (by system):* General: cytokine release syndrome, fever, febrile neutropenia; Cardiovascular: hypotension, tachycardia; CNS: neurotoxicity; (e.g., confusion, delirium, dysgraphia, dysphasia, seizures) Endocrinopathies: electrolyte derangements; Gastrointestinal: nausea, vomiting, diarrhea, abdominal pain; Hematology/Immunology: cytopenias, hypogammaglobulinemia; Hepatic: transaminitis, hyperbilirubinemia; Renal: acute renal failure; Respiratory: hypoxia, cough, dyspnea, tachypnea
**Immune checkpoint inhibitor**
*Mechanism of Action*
Immune checkpoint inhibitor: Disrupt signaling pathways (either from tumor suppression, or immune checkpoint proteins) that suppress T cell function, enabling T cells to provide an enhanced immune response against tumor cells
*Toxicity Profile (by system):* General: fevers; Cardiovascular: cardiac arrhythmias, peripheral edema; CNS: fatigue, pain, headache, myalgias, arthralgias, asthenia; Dermatologic: rash, pruritis; Endocrinology: electrolyte derangements, hypophysitis, hypo- hyper-thyroid, myxedema; Gastrointestinal: abdominal pain, diarrhea, constipation, decreased appetite, nausea, vomiting; Genitourinary: hematuria, urinary tract infection; Hematologic: cytopenias; Hepatic: transaminitis; Immune related adverse events: colitis, hepatitis, myocarditis, pneumonitis; Infection: infection; Renal: increased serum creatinine; Respiratory: cough, dyspnea, pneumonia, upper respiratory infection
**Antibody based therapy**
*Category Type and Mechanism of Action*
Bispecific T cell engager: Immunotherapy that is a bispecific antibody; designed to target CD3 and tumor-specific antigens simultaneously and promote the cytotoxicity of T cells.
Monoclonal antibody: Immune system proteins that are synthetic and enable your immune system to recognize and destroy cancer cells either by binding a specific antigen that alerts the immune system to attach a cancer cell, or by forming a link between T cells and tumor cell with antigen specificity and enables T cells to exert cytotoxic activity on tumor cells.
Antibody-drug conjugate: Targeted agents that link a cytotoxic drug or molecule to a monoclonal antibody which then specifically targets a cell surface antigen, binds and becomes internalized. Once internalized an endosome is formed and fuse with lysosomes which then are cleaved, resulting in cytotoxic drug or molecule to be released into the cytoplasm and cause apoptosis or cell death.
*Toxicity Profile (by system):* General: flu-like symptoms, fever, infusion reactions, angioedema, cytokine release syndrome; Cardiovascular: flushing, hypertension, hypotension, peripheral edema, thromboembolic events, capillary leak syndrome, tachycardia; CNS: neurotoxicity (e.g., confusion, delirium, dysgraphia, dysphasia, seizures), fatigue, headache, pain, insomnia, arthralgias, anxiety, dizziness, myasthenia; peripheral neuropathy, insomnia; Dermatologic: night sweats, pruritis, rash, desquamation, dermatitis, paronychia, alopecia, urticaria; Endocrinology: electrolyte derangements, weight loss or gain; Gastrointestinal: abdominal pain, diarrhea, nausea, vomiting, dysgeusia; Genitourinary: urinary tract infection; proteinuria, pelvic pain; Hematology/Immunology: cytopenias, bruising, hypogammaglobulinemia, antibody development; Hepatic: hepatobiliary disease, transaminitis, sinusoidal obstruction syndrome; Infection: infection, bacterial most common; Respiratory: bronchitis, cough, pulmonary disease, pulmonary hemorrhage
**Vaccines**
*Mechanism of Action*
Vaccines: Exogenous administration of selected tumor antigens combined with adjuvants to help stimulate the immune system such as dendritic cells. The aim of vaccine therapeutics is to stimulate the adaptive immune system against specific tumor antigens to regain control over tumor growth. This can be done by inducing large trafficking of dendritic cells to tumor sites, induction and maintenance of sustained T cell response, and infiltration of the tumor microenvironment.
*Toxicity Profile (by system):* CNS: headaches, edema at tumor sites, seizures, hemiparesis, dysphasia, confusion; Immune-related events: injection site reaction, fever, rash, pruritus, fatigue, chills; Hematologic: neutropenia, lymphopenia; Hepatic: transaminitis;

Utilization of immunotherapy in pediatric oncology continues to expand, necessitating both an enhanced understanding of how irAEs may manifest and how management strategies may differ in younger children. While generalized irAE or immune effector cell related toxicity management guidelines are increasingly available (11–14), experiences are predominantly based on treatment of adults. In the specific context of neurotoxicity, both the evaluation for and presentation of neurotoxicity may be quite disparate between children and adults, particularly in very young children. Treating clinicians must have both the knowledge of the neurotoxicities associated with specific immunotherapies and carry a high index of suspicion in the evaluation of patients with a new neurologic finding. In this review, we will focus on providing an overview of the neurotoxicity profile of the most used immunotherapeutic approaches in children and young adults and how they may manifest across the spectrum of pediatric cancer diagnoses.

## Pediatric Specific Considerations for Evaluation of Immunotherapy Associated Neurotoxicity

The most important clinical consideration in children is the difference in approach toward recognition of neurotoxicity. Some tools that are used for detecting neurotoxicity in adults rely largely on language function and cannot be used for young children (15). Mild or early neurotoxicity may manifest in subtle behavioral changes that are only recognized by caregivers who know the child well. Therefore, prospective monitoring systems must be in place to catch early signs and symptoms. One excellent tool, the Cornell Assessment of Pediatric Delirium (CAPD), was validated for recognizing delirium in children hospitalized in intensive care units (16). This tool has been adopted into the guidelines for neurotoxicity monitoring after CAR T cell therapy and is now commonly used in settings where it has not yet been validated, such as acute care units and even outpatient care (15). A custom caregiver checklist was also developed to monitor pediatric and young adult patients for neurotoxicity in the acute setting post-CAR T cell therapy (17).

Apart from behavioral changes, several other signs and symptoms deserve special consideration in the pediatric population. Headache alone may or may not be considered a true neurotoxicity, yet it can be a harbinger of more serious CNS pathology. Headache can be difficult to ascertain in very young children, who may present instead with irritability. Changes in motor strength often go unrecognized in ill young children because of their limited ability to participate in a direct confrontational exam. Other unique considerations in pediatrics include the type of workup required for neurotoxicity, as children may require sedation for MRI, which can delay diagnosis, and may not be able to tolerate prolonged electroencephalography (EEG) monitoring or nerve conduction studies, hampering our ability to detect subtle toxicities which may be underrecognized.

Involvement of neurology consult services should occur prospectively, particularly in patients who have preexisting neurologic comorbidities, those with neurodevelopmental differences (18), or those receiving therapy at high risk of causing neurotoxicity. This is particularly important in younger children who require specialized neurologic exam techniques to account for developmental age. Baseline neurologic examination including detailed neurologic past medical history should be performed prior to therapy, or as soon as suspicion for neurotoxicity is raised.

There is no evidence currently to suggest differences in the mechanism of neurotoxicity-associated with immunotherapy between children and adults. Indeed, toxicities of CD19-directed CAR T cell therapy have been well studied in both children and adults with ALL, with similar rates of neurotoxicity and CRS (19). Nonetheless, for some agents there are differences in the reported incidence of specific subcategories of neurotoxicity between children and adults (**Table 2**).

**Table 2:** Comparison of Neurotoxicity in FDA Approved Immunotherapy agents in adults or pediatrics.

Drug Name FDA Approval Status	Target	Incidence and Type of Neurotoxicity AEs in Adults	Incidence and Type of Neurotoxicity AEs in Pediatrics	References for Pediatric Studies
**Adoptive cell therapy**				
Tisagenlecleucel	CD19	Anxiety 9%	Anxiety 13%	PMID: 25317870
FDA Approved:		Delirium 6%	Delirium 21%	PMID: 29385370
Pediatrics and Adults		Dizziness 11%	Dizziness 6%	
		Encephalopathy 16%	Encephalopathy 34%	
		Headache 21%	Headache 37%	
		Peripheral neuropathy 8%	Peripheral neuropathy 4%	
		Sleep disorders 9%	Sleep disorders 10%	
		Tremor 7%	Tremor 9%	
**Immune checkpoint inhibitor**				
Atezolizumab	PD-L1	Arthralgia 12-13%	Headaches 19.5%	PMID: 31780255
FDA Approved:		Asthenia <52%	Meningoencephalitis 1.1%	
Adults		<1%: Demyelinating disease, encephalitis, exacerbation of myasthenia gravis, Guillain-Barre syndrome, meningitis, myasthenia gravis, neuropathy, and paresis	Cranial nerve disorder 1.1%	
			Hydrocephalus 1.1%	
			Migraine 1.1%	
			Neuralgia 2.2%	
			Papilledema 1.1%	
			Paresthesia 1.1%	
Ipilimumab	CTLA-4	Headaches 15-33%	Headache 3%	PMID: 26534966
FDA Approved:		Insomnia 10%	Vision changes 3%	PMID: 25416723
Adults and Pediatric > 12 years old with certain cancers		Neuropathy 1.7%		
		<1%: Demyelinating disease, encephalitis, Guillain-Barre syndrome, meningitis, myasthenia gravis, motor dysfunction, and nerve palsy		
Nivolumab	PD-1	Dizziness 11%	Headaches 20%	PMID: 32192573
FDA Approved:		Headache 23%	Paresthesia 7.7%	PMID: 33892407
Adults and Pediatric > 12 years old with certain cancers		Arthralgia 20%		
		Asthenia up to 57%		
		Pain 32-42%		
Pembrolizumab	PD-1	Altered mental status 3%	Arthralgia 10.4%	PMID: 31812554
FDA Approved:		Arthralgia 10-18%	Asthenia 12.3%	
Adults and Pediatric > 12 years old with certain cancers		Asthenia: 10-11%	Ataxia 1.2%	
		Confusion 2%	Balance disorder 0.6%	
		Dizziness 5%	Blindness 0.6%	
		Headache 11-14%	Depressed LOC 0.6%	
		Insomnia 7%	Headaches 22%	
		Myalgia 12%	Hemiparesis 2.6%	
		Pain 22%	Neuralgia 1.2%	
		Peripheral neuropathy 1-11%	Opisthotonos 0.6%	
		<1%: Demyelinating disease, encephalitis, exacerbation of myasthenia gravis, Guillain-Barre syndrome, meningitis, myasthenia gravis, neuropathy, and paresis	Seizures 4.5%	
			Spinal cord compression 1.2%	
			Stroke 0.6%	
			Tremor 1.2%	
			Vision blurred 3.2%	
**Antibody based therapy**				
**Bispecific T-cell engager**				
Blinatumomab	CD19	Aphasia <12%	Agitation 7.4%	PMID: 33651091
FDA Approved: Pediatric and Adults		Dizziness <10%	Anxiety 3.7%	PMID: 33651090
		Encephalopathy 2-10%	Dizziness 1.9%	PMID: 32709851
		Headaches 23-39%	Encephalopathy 1.9-15%	PMID: 27998223
		Seizure >2%	Headache 25-35.2%	
		Tremor < 31%	Neuralgia 1.9%	
			Seizures 3.7-5%	
			Tremor 9.3%	
**Monoclonal antibody**				
Bevacizumab	VEGF-A	Anxiety 17%	Pain 40%	PMID: 31967673
FDA Approved:		Arthralgia 28-41%	Neuropathy 7.7-8.3%	PMID: 23894304
Adults		Asthenia (grade 3/4) 10%	Reversible posterior	PMID: 23630159
		Blurred vision 2%	leukoencephalopathy syndrome 5.9%	
		Dizziness 23%	Seizure 5.9%	
		Dysarthria 8-12%	Speech impairment 6.6%	
		Headache 22-42%		
		Insomnia 21%		
		Myalgia 19%		
		Myasthenia 13-15%		
		Pain (grade 3/4) 8%		
		Voice disorder 5-13%		
		<1% Reversible posterior leukoencephalopathy syndrome		
Cetuximab	EGFR	Anxiety 14%	Headaches 56%	PMID: 19770383
FDA Approved:		Arthralgia 14%		
Adults		Asthenia <73%		
		Confusion 18%		
		Depression 14%		
		Headache 19-38%		
		Insomnia 27%		
		Malaise <73%		
		Pain 59%		
		Peripheral neuropathy 45%		
Dinutuximab	GD2	Not FDA approved in Adults	Blurred vision 2%	PMID: 28549783
FDA Approved:		Severe motor neuropathy 13%	Pain 85%	PMID: 32343642
Pediatrics		Other side effects unknown	Peripheral neuropathy 13%	PMID: 31815885
			Peripheral motor neuropathy 1%	PMID: 26791869
			Peripheral sensory neuropathy 9%	PMID: 11118469
				PMID: 23924804
				PMID: 11118469
				PMID: 19047298
				PMID: 9626218
				PMID: 20879881
				PMID: 7718335
Rituximab	CD20	Anxiety 2-5%	Central neurotoxicity 8.8%	PMID: 20516455
FDA Approved:		Arthralgia 6-13%	Cerebellar syndrome 0.6%	
Adults		Asthenia 2-26%	Chorioretinitis 0.6%	
		Dizziness 10%	Confusion 0.6%	PMID: 32492302
		Fatigue 13-39%	Headaches 21%	
		Headaches 17-19%	Meningitis 0.6%	
		Myalgia 10%	Peripheral neurotoxicity 2.9%	
		Pain 12%		
		Paresthesia 2%		
		Peripheral sensory neuropathy 30%		
**Antibody-drug conjugate**				
Brentuximab vedotin	CD30	Arthralgia 12-19%	Arthralgia 6%	PMID: 30290902
FDA approved: Adults		Asthenia 11%	Headache 8%	PMID: 33826362
		Dizziness 11-16%	Myalgia 14%	
		Fatigue 24-49%	Pain 4-8%	
		Headache 11-19%	Paresthesia 19%	
		Myalgia 11-17%	Peripheral neuropathy 4-33%	
		Neuropathy 62%	Peripheral sensory neuropathy 11%	
		Pain 28%		
		Peripheral motor neuropathy 16-23%		
		Peripheral sensory neuropathy 45-56%		
Inotuzumab ozogamicin	CD22	Fatigue 35%	Headaches 28%	PMID: 30267011
FDA Approved: Adults		Headaches 28%		PMID: 33067614

## Neurotoxicity Considerations by Oncologic Diagnosis

### Hematologic Malignancies

Hematologic malignancies in pediatrics are the most diagnosed pediatric cancers and include acute and chronic leukemias, and both Hodgkin and Non-Hodgkin lymphomas (20). In general, pediatric patients with hematologic malignancies have a good prognosis, with 5-year overall survival rates ranging from 70-95% based on diagnosis (21–34). Improvement in outcomes have been noted over the last several decades in part due to cooperative group trials that incorporated multi-drug chemotherapy, early risk stratification with intensification or de-intensification therapy, implementation of CNS prophylaxis, and improvement in supportive care measures.

Despite the remarkable success rates in treating pediatric hematologic malignancies, these patients are at high risk of neurologic sequelae as a result of multi-modal therapy with systemic chemotherapy, repetitive intrathecal therapy, radiation therapy (either with total body irradiation or CNS directed therapy), and hematopoietic stem cell transplantation. Neurocognitive deficits are common, especially if therapy is administered early in childhood (35, 36). Additionally, these neurocognitive adverse events (nAEs) may manifest years after completion of therapy.

Given that immunotherapy is currently used in relapsed/refractory disease, the impact of prior therapy is important to note, as patients may already have neurologic comorbidities that may impact the patients’ ability to tolerate additional neurologic insults or leave them more vulnerable to neurotoxicity from additional therapy. As timing of immunotherapy continues to evolve with more trials moving it into the upfront treatment setting, immunotherapy may represent an attractive opportunity to decrease neurologic sequelae in this patient population.

### Solid Tumors

Pediatric solid tumors located outside the central nervous system (CNS) comprise 20% of all newly diagnosed pediatric cancers. This broad category is composed of sarcomas, neuroblastoma, Wilms tumor, hepatoblastoma, germ cell tumors and other rare malignancies (37). While the overall cure rates for childhood cancer have improved significantly over the last 40 years with the use of multimodal therapy including surgery, chemotherapy, and radiation, improvements in outcome for children with solid tumors have lagged behind progress seen in hematologic malignancies (38). Children with high-risk, metastatic or recurrent disease face high mortality rates and new treatment modalities, including immunotherapy, are being actively evaluated. Nearly all children and young adults who receive chemotherapy and radiation will endure late sequelae of current curative treatments. Specifically, neurologic toxicity from therapy can include epilepsy, peripheral neuropathies, and impairment of vision and hearing (39–41). Novel treatment approaches, including immunotherapy, may ultimately impact survival and can reduce late effects of current treatment methods. Early phase clinical trials conducted by cooperative groups like Children’s Oncology Group facilitate the study of novel treatments in relatively rare patient populations. One notable example of how immunotherapy has changed the treatment paradigm for a high-risk pediatric solid tumor is the success of the anti-GD2 antibody, dinutuximab, which is FDA approved as part of first line therapy for high-risk neuroblastoma patients (42). The growing field of immunotherapy for solid tumors include T cell checkpoint inhibitors, monoclonal antibodies, antibody-drug conjugates, genetically engineered immune cells and vaccines. Unique neurotoxicity considerations for patients with solid tumors are largely attributed to the immunotherapy itself as opposed to the site or extent of disease.

### Central Nervous System Tumors

Primary tumors of the CNS are the second most diagnosed pediatric malignancy, and the leading cause of cancer death in the pediatric population (43, 44). Approximately 550 children die from brain tumors annually in the US, the majority from high grade gliomas (HGG), medulloblastomas, and atypical teratoid rhabdoid tumors (44, 45). Treatment for CNS tumors typically consists of chemotherapy, radiation, and/or surgery, which commonly leaves survivors with debilitating morbidity and devastating neurologic sequelae including neurocognitive delays, neuroendocrine abnormalities, sensory disturbances, hearing loss, and seizure disorders (46–48).

Immunotherapy for pediatric brain tumors is evolving rapidly as a promising new treatment that can potentially improve both morbidity and neurocognitive outcomes in survivors (49–51). Importantly however, several key considerations including route of delivery and toxicity profile for CNS-targeted immunotherapies need to be investigated to optimize immunotherapy for childhood CNS tumors. In contrast to solid and hematologic malignancies, where systemic delivery of therapy has demonstrated the ability of treatment to reach sites of disease, the unique immune privilege of the CNS and the role of the blood-brain-barrier (BBB) need to be investigated to determine the ideal route of administering novel therapies. Additionally, tumor-targeting immune activity is likely to induce focal inflammation and edema in the CNS, which can cause new or worsening neurologic signs and symptoms such as weakness or focal seizures.

Another major challenge in CNS tumor immunotherapy is how to distinguish irAEs (also known as immune-related pseudoprogression) from tumor progression, which is important for ongoing medical treatment management. There are currently no imaging techniques that can reliably distinguish immune-related pseudoprogression from tumor growth in the acute setting (52). In 2015, immunotherapy Response Assessment for Neuro-Oncology (iRANO) MRI criteria were developed to account for possible immune-related pseudoprogression (53). However, the iRANO criteria have not yet been extensively validated, and will likely require further refinement (54, 55). When immune-related pseudoprogression is suspected, steroids are typically the first-line therapy, but clinical responses to steroids are similar for treatment-related and tumor-related edema and inflammation.

In addition to primary CNS tumors, brain metastases from non-CNS primary solid tumors and hematologic malignancies also cause significant morbidity. Brain metastases occur in approximately 1.4-4.9% of children with solid tumors (56–60), and approximately 10-20% of children with leukemia/lymphoma (61). Despite their high potential for morbidity, brain metastases are particularly poorly studied in the field of pediatric cancer immunotherapy. Toxicities from immunotherapies would be expected to be similar as for primary CNS tumors (local immune activation within the CNS), with possible overlap of neurotoxicity related to systemic immune activation.

## Neurotoxicity Manifestations by Immunotherapeutic Treatment Approach

### Adoptive Cell Therapy

#### General

Adoptive cell therapy uses immune cells to target cancer epitopes, either by selecting tumor-targeting immune cell subsets, or by genetically modifying them. The current most successful modality, chimeric antigen receptor (CAR) T cells, uses the latter approach. CARs are synthetic molecules that combine the antibody recognition properties (non-MHC restricted) of a B cell and the effector functions of a T cell. They consist of two domains, an extracellular binder (typically a single chain variable fragment) against a specific cell surface tumor epitope, and an intracellular signaling domain that induces target killing and T cell proliferation (62–66). The CAR transgene is introduced into autologous or allogeneic T cells, enabling them to identify and eliminate any cells that express the target antigen. This includes tumor cells (on-target/on-tumor activity) and normal cells expressing the target epitope (on-target/off-tumor activity). In addition, CAR T cell activity and expansion is typically accompanied by significant systemic cytokine release (62–66). Other types of adoptive cell therapy that have been tested in children include genetically modified T cell receptors (TCRs) and Natural Killer (NK) cell therapy. Bispecific antibodies, which serve as a linker between immune cells and cancer targets, are covered in the antibodies section of this review.

#### Hematologic Malignancies

CAR T cell therapy has revolutionized cancer treatment by inducing durable remissions in pediatric patients with relapsed/refractory B cell leukemia (B-ALL). Several studies have been published using CAR T cells for treatment of pediatric patients with relapsed/refractory B cell hematologic malignancies and have demonstrated impressive complete remission rates of 60-90% (67–72). The pivotal phase II global registration trial for CD19 targeted CAR T cells led to the historic, first pediatric FDA approved CAR product, tisagenlecleucel, for children and young adults up to age 25 with relapsed/refractory B-ALL (68, 73). Common toxicities (incidence > 20%) that occur post-CAR therapy in pediatric patients with B-ALL include CRS, neurotoxicity, infection, hypogammaglobulinemia, and fatigue (67–72, 74).

#### Neurotoxicity Experience in CAR T Cells

Neurotoxicity, now known as immune effector cell-associated neurotoxicity syndrome (ICANS) (15), is a common adverse event associated with CAR T cell therapy, with variable incidence of 7%-72% in published trials using CD19, CD22, or CD19/22 CAR T cells in children and young adults with hematologic malignancies (67–72, 74–77). In a recently published donor-derived CD7 CAR T cell trial for T cell ALL (T-ALL), incidence of ICANS was 15%, and all cases were grade 1 and associated with CNS disease (78). More severe cases of ICANS (≥ grade 3) have been described in up to 20% of pediatric patients treated with CAR T cell therapy. Clinical manifestations of ICANS vary, with most common presentations including depressed levels of consciousness, confusion, headache, tremors, and language disturbances (67–72, 74–77). More severe presentations of encephalopathy, seizures, and coma have occurred in up to 20% of patients, with rare cases of fatal cerebral edema (77, 79, 80). The incidence of seizures also varied significantly across studies, 0-20%, with most seizures being generalized tonic-clonic (67–72, 74–77). ICANS is often preceded by CRS onset, typically presenting within 7 days post-CAR infusion.

Acute neurocognitive functioning and patient reported outcomes measures have also been evaluated in pediatric patients receiving CAR therapy. Data collection in this patient population is feasible, with stable neurocognitive function and clinically meaningful health-related quality of life improvements demonstrated over time (17, 81).

As the application for CAR T cells continue to broaden, so too may the toxicity profile. Several hematologic CAR T cell trials in pediatrics are ongoing including those targeting CD5 and CD7 in T-ALL, and CD33 and CD123 in AML. Additionally, combinatorial CAR constructs with CD19, CD22, and/or CD20 are currently in clinical trials.

#### Pathophysiology of ICANS

The pathophysiology of ICANS post-CAR therapy remains under active investigation. Endothelial cell activation, vascular and blood brain barrier disruption, vascular leak, systemic inflammatory response with CRS, and off-tumor/on-target toxicity are all possible mechanisms by which neurotoxicity can occur in pediatric patients (10, 76, 77, 82, 83). Indeed, the highest risk of ICANS is typically seen during the acute CAR T cell expansion, which suggests that the systemic inflammatory surge drives cytokine mediated neurotoxicity, rather than a local CNS cytokine production (77). Mouse models have suggested possible roles for IL-1 and GM-CSF signaling underlying the development of neurotoxicity (84, 85).Additionally, this systemic inflammation can also lead to endothelial activation, astrocyte injury, and increased permeability of the blood brain barrier (76, 77). However, an association of CAR T neurotoxicity with elevated Angiopoietin-2 levels, indicative of endothelial activation, was shown in adults (10) but not children (77, 86). Increased CSF glial fibrillary acidic protein (GFAP), and S100 calcium-binding protein (S100b), were found to be elevated in the acute setting post-CAR, indicating astrocyte injury and activation, which could lead to osmotic dysregulation in the brain potentially causing or contributing to cerebral edema (77, 83). In a mouse model, blockade of brain capillaries by circulating leukocytes was associated with the development of neurotoxicity (87).CAR T cell trafficking to the CNS has been demonstrated in several pivotal pediatric trials, but presence or quantity of CAR T cells in the CSF does not predict whether ICANS will occur (67, 68, 70, 88, 89). In fact, CAR T cells are detected in the CSF of patients with and without ICANS. Recently, a study demonstrated CD19+ mural cells surrounding the endothelium of the brain, suggesting that off-tumor/on-target toxicity of CD19 CAR T cells can contribute to neurotoxicity (90). However, this finding needs to be cautiously interpreted as ICANS is seen with CAR T cells targeting other antigens (B cell maturation antigen, or CD22) and not all patients who receive CD19 directed CAR T cells develop neurotoxicity.

#### Risk Factors for CAR T Cell ICANS

The presence and severity of cytokine release syndrome is the strongest and most consistent risk factor for ICANS in pediatric CAR T cell patients (68, 76, 77, 91). There may also be additional risk conferred by pre-existing neurologic comorbidities (76, 91), including abnormal baseline brain MRI (77). Differences in demographics, CNS disease status, treatment history including prior brain radiation and bone marrow transplant, bone marrow disease burden at the time of CAR infusion, lymphodepletion regimen, or CAR T cell dose was not associated with development of ICANS in pediatric patients (70, 77, 91). It is important to note that disease burden, lymphodepletion regimen, and CAR T cell dose are associated with the development of ICANS in adult patients with hematologic malignancies, which likely affect the incidence and severity of CRS (10, 92, 93).

#### Evaluation and Management of CAR T Cell Related ICANS

The American Society for Transplantation and Cellular Therapy (ASTCT) grading criteria are now used to grade ICANS (15). ICANS grading incorporates a 10-point encephalopathy score for children >12 years and adults (ICE) or the CAPD score for children <12 years, as well as additional neurologic domains including level of consciousness, seizures, motor symptoms, and signs of increased intracranial pressure (ICP) or cerebral edema. Grading is typically based on the most severe symptoms present in each category. Treating medical teams need to perform detailed past medical histories including evaluation of any prior treatment-related neurologic toxicities and neurologic risk factor comorbidities (e.g., seizure disorder) in each patient prior to receipt of CAR T cells. Baseline neurologic examination should also include ICE or CAPD scores. In patients with a history of neurologic adverse events, CNS disease, or focal findings on exam, baseline neuroimaging should be considered. Additionally, for those deemed high-risk (e.g., those who have a prior history of seizures), or patients receiving CAR products that have high neurotoxicity incidence, anti-seizure prophylaxis prior to receipt of CAR T cells should be initiated. Post-CAR T cell infusion, patients should be monitored for the development of neurotoxicity with routine neurologic exams inclusive of ICE or CAPD scores, especially once cytokine release syndrome has started. In patients who develop ICANS, work up should be performed based on the severity of symptoms present, with routine labs, neurology consult, neuroimaging, EEG, and evaluation for infectious etiologies, with consideration for a lumbar puncture if deemed safe (13).

Based on the clinical data that has emerged from CD19-directed therapy in pediatric and young adult patients receiving CAR T cells, management of ICANS remains largely supportive, with administration of anti-pyretics and/or antiseizure medications. Although tocilizumab has demonstrated remarkable results in abrogating CRS severity, it has not proven beneficial in treatment of ICANS (10, 67, 68, 77, 92, 94). Based on ASTCT grading criteria, use of corticosteroids for ICANS should be considered in patients with grade 2 ICANS, and is recommended for patients who have grade 3 or grade 4 ICANS (13), with consideration of intrathecal hydrocortisone administration in cases of severe ICANS (95). Additionally, in patients with seizures, levetiracetam is recommended (96). When there is suspicion for worsening ICANS or signs of increased ICP, patients should be transferred to the ICU for close monitoring and initiation of urgent management as clinically indicated. In a majority of pediatric ICANS cases, symptoms typically resolve by day 28, however, rare chronic neurologic sequelae and fatal cases have been reported (77).

#### Solid Tumors

There are many ongoing adoptive cell therapy trials for solid tumors utilizing a patient’s own T cells or NK cells to directly target tumor cells. There are currently published results from clinical trials of CAR modified T cells or NK cells targeting CD171/L1CAM, GD2, and HER2 in pediatric patients with solid tumors (97–99, 102). All trials thus far show feasibility and safety of infusing CAR modified T cells or NK cells, with no obvious neurotoxicity. GD2 and CD171/L1CAM are both proteins known to be expressed by cells in the central nervous system and peripheral nerves, but no overt toxicities have been observed in clinical trials targeting these proteins (96–101). There is one published study of T cells genetically engineered with an NY-ESO-1 reactive T cell receptor (TCR) that includes young adults with synovial sarcoma, where antitumor efficacy was demonstrated and no neurotoxicity was seen (103). Of note, Guillain-Barre syndrome has been observed in adults treated with NY-ESO-1 directed T cell receptor therapy (104).

#### CNS Tumors

Multiple pediatric CNS tumor CAR T cell trials are underway, as well as trials using tumor specific T cells, but toxicity data is just beginning to emerge (105). Common to all studies is that focal neurologic symptoms were frequently seen after CAR T cell treatment for brain tumors, but typically the authors were not able to definitively distinguish progressive disease from immune-mediated toxicity. Results from two HER2-targeting trials showed a good safety profile. In a mixed age trial of HER2-CAR transduced virus specific T cells for glioblastoma that included 7 children, one child had a neurologic adverse event. This was one of the two patients among the 17 total patients who developed seizures and/or headaches (106). In a study of intracranially delivered HER2 CAR T cells, 3 of 3 patients developed headache and/or back pain. One patient had transient worsening of baseline neurologic deficits after the first CAR T cell dose, which was accompanied by increased peritumoral edema and contrast enhancement on imaging. Although this constituted a possible immune response, the patient had confirmed progressive disease 2 months after initiating treatment and did not meet iRANO criteria for pseudoprogression (107). A trial of GD2-targeting CAR T cells for diffuse intrinsic pontine gliomas and spinal cord diffuse midline gliomas showed evidence of efficacy, and evidence of intratumoral inflammation which the authors termed Tumor Inflammation-Associated Neurotoxicity (TIAN) (105, 108). Additional trials targeting HER2, GD2, B7H3, EGFR, and IL13Rα2 are ongoing.

### Immune Checkpoint Inhibitors

#### General

Immune checkpoint proteins are a part of the adaptive immune system and their role is to prevent the immune system from over-engaging and destroying healthy cells (109). Under normal conditions, inhibitory checkpoint proteins work by engaging and binding to partner proteins and often initiate signaling pathways that suppress T cell function. Sometimes, however, cancer cells can evade detection by simulating immune checkpoint proteins thereby inhibiting T cell mechanics. Drugs that block the inhibitory checkpoint proteins, known as immune checkpoint inhibitors (ICIs), target immune checkpoints and can overcome tumor-mediated inhibition of T cell function. This effectively restores immune system function and often leads to T cell activation and tumor killing. ICIs have revolutionized cancer treatment paradigms and are approved for the treatment of multiple adult and pediatric malignancies (110, 111). With disruption of the checkpoint pathway, effectively taking the brakes off the immune system, ICIs can lead to a spectrum of inflammatory side effects including gastrointestinal, dermatologic, and endocrine toxicities (112). In the adult population, the incidence of neurologic complications ranges from 2-4% (1). In large pharmacovigilance studies, the most commonly reported neurologic syndromes were inflammatory myopathies, myasthenia gravis, noninfectious encephalitis or meningitis, and peripheral neuropathy (113, 114). Relapses of preexisting autoimmune neurologic conditions such as multiple sclerosis can occur (115, 116).

### Neurotoxicity Experience in Immune Checkpoint Inhibitors

#### Hematologic and Solid Tumor Malignancies

Use of checkpoint inhibitors in pediatric clinical trials often includes patients with different oncologic diagnoses including hematologic (Hodgkin’s and Non-Hodgkin’s lymphoma) and solid tumor malignancies, with few cases reported in leukemia (117–121).

The overall reported incidence of neurotoxicity across trials ranges from 20-53%, with headaches being the most common neurologic symptom reported (118, 120, 121). Other neurologic side effects that occurred less commonly included blurred vision, seizures, and hemiparesis, with reports of severe toxicity such as stroke, blindness, encephalitis, and spinal cord compression occurring at approximately 1% incidence (120, 121). (See **Table 3** for neurotoxicity related to individual agents in the larger pediatric clinical trials). Additionally, hypophysitis, which is a common irAE (122), can develop in patients treated with ICIs and often presents with concurrent neurologic symptomatology. Thus, assessment of endocrine function should always be part of the evaluation of neurologic complications of checkpoint blockade.

**Table 3:** Select Pediatric Clinical Trials with Specific Neurotoxicity Descriptions.

Drug Name	Target/MOA	Subjects treated (n)	Neurotoxicity (incidence, % if known)	PMID
**CAR T cells**				
Tisagenlecleucel	CD19-directed CAR T cell	n=51	Headache (36%)	PMID: 25317870
		n=75	Encephalopathy (11-37%)	PMID: 29385370
			Focal deficits (28%)	PMID: 30178481
			Confusion (9%)	
			Delirium (9%)	
			Tremor (8%)	
			Agitation (7%)	
			Seizure (1.3-8%)	
CD19 CAR T cells	CD19-directed CAR T cell	n=50	Altered mental status (32-48%)	PMID: 33764809
		n=43	Delirium (35%)	PMID: 31074527
		n=25	Headache (20-21%)	PMID: 31650176
		n=74	Tremor (19-28%)	PMID: 34156874
			Decreased level of consciousness (16%)	
			Seizure (4-20%)	
			Involuntary movements (8%)	
			Ataxia (8%)	
			Dysphasia (4%)	
**Immune checkpoint inhibitor**				
Nivolumab	Human immunoglobulin G4 (IgG4) monoclonal antibody that binds to the PD1 receptor and blocks its interaction with PDL-1 and PDL-2	n=85	Headaches (20%)	PMID: 32192573
			Grade 3 CNS dysfunction (5%)	
Pembrolizumab	Humanized monoclonal antibody that binds to the PD-1 receptor and directly blocks the interaction between PD-1 and PD-L1/PD-L2	n=154	Reported grade 1-2 nAEs (82%):	PMID: 31812554
			headaches, blurred vision, asthenia, and hemiparesis	
			Reported > grade 3 nAEs (18%):	
			blindness, blurred vision, ataxia, balance disorder, hemiparesis, ischemic stroke, neuralgia, opisthotonos, tremor (all 0.6%); seizures, headaches, and spinal cord compression (all 1.3%)	
Atezolizumab	Humanized immunoglobulin G1(IgG1) monoclonal antibody that blocks PD-L1	n=87	Grade 1-2 nAEs:	PMID: 31780255
			Headaches (19.5%)	
			Meningoencephalitis (1.1%) Reported > grade 3 nAEs: cranial nerve VI disorder, headache, hydrocephalus, migraine, papilledema, paresthesia (all 1.1%); neuralgia (2.3%)	
**Antibody-Based Therapy:**				
**Bispecific T cell Engager**				
Blinatumomab	Designed to target CD3 and CD19 simultaneously and promote the cytotoxicity of T cells	n=70	Headache (30-35%)	PMID: 27998223
		n=105	Pain (11-20%)	PMID: 33651090
		n=54	Encephalopathy (2-15%)	PMID: 32094465
			Tremor (6-9%)	
			Agitation (7.4%)	
			Arthralgia (6%)	
			Muscle weakness (6%)	
			Seizures (3.7-5%)	
			Dizziness (4%)	
**Monoclonal antibody**				
Epratuzumab	Binds extracellular CD22, and is rapidly internalized, and functions by altering and modulating B-cell activation and signaling	Phase 1: n=15	Rare neurotoxicity was observed: seizure (6.7%);	PMID: 18669463
		Phase II: n=114 (54 received weekly dosing, 60 received twice weekly dosing)	Neurotoxicity was more commonly seen in patients receiving twice weekly dosing, albeit rare in both cohorts: peripheral neuropathy, anxiety (both 1.85%); cognitive disturbances, dizziness, dysphagia, headache, intracranial hemorrhage (all 1.67%); encephalopathy (3.3%)	PMID: 25732247
Rituximab	Monoclonal antibody that targets CD20 and induces both complement-mediated cytotoxicity and antibody-dependent cell-mediated cytoxicity	Phase II: n=136	Most commonly reported nAEs included pain, with headaches accounting for 21%; central (8.8%) and peripheral (2.9%) neurotoxicity. Myalgias and arthralgias were also described	PMID: 20516455
		Phase III: n=164	Few grade 4 nAEs <1%: chorioretinitis, cerebellar syndrome, confusional state, and meningitis	PMID: 32492302
Bevacizumab	Humanized monoclonal antibody that acts by blocking angiogenesis via neutralizing free circulating VEGF-A.	n=105	Reported cases of intracranial hemorrhage varies by trial (6-11%)	PMID 33844469
		n=35		PMID: 24311632
		n=22		PMID 25859842
		n=27	CNS ischemia (2.9-6.5%)	PMID 26626490
				PMID: 32556862
				PMID: 20479404
	It is commonly used in combination with chemotherapy and/or radiation.	n=38	Several pediatric clinical trials for brain tumors have reported no neurologic adverse events	PMID: 33963476
				PMID: 34359048
		n=36		PMID: 31967673
		n=31		
		n=9	Pain: (40%)	
		n=13	Speech impairment*: (7%)	
		n=15	*In combination with everolimus	
Cetuximab	Chimeric monoclonal antibody that binds epidermal growth factor receptor	n=13	No nAEs reported in brain tumors	PMID: 34359048
		n=46		PMID: 19770383
			Headache*: 56%	
			*In combination with irinotecan in solid tumors	
Nimotuzumab	Humanized monoclonal antibody that binds epidermal growth factor receptor	n=25	No nAEs when combine with radiation and vinorelbine in patients with DIPG	PMID: 24696052
		n=42	Headaches occurred in 7% of patients who were treated with nimotuzumab plus radiation in DIPG	PMID: 30830679
			Intracranial hemorrhage	
			Neurologic deterioration	
Dalotuzumab	Insulin-like growth factor receptor directed humanized monoclonal antibody	n=24	Abdominal pain: (10%)	PMID: 27185573
Cixutumumab	Insulin-like growth factor receptor directed humanized monoclonal antibody	n=39	Headache: 1-18%	PMID: 25467181
		n=114	Oral cavity pain: 2%	PMID: 23956055
		n=47		PMID: 22184397
RG1507	Insulin-like growth factor receptor directed human monoclonal antibody	n=31	Pain: 25%	PMID: 21127194
Lexatumumab	Tumor necrosis factor–related apoptosis-inducing ligand receptor 2 directed human monoclonal antibody	n=24	Pain: Described as common	PMID: 23071222
Ontuxizumab	Endosialin directed humanized monoclonal antibody	n=22	Headache: 27%	PMID: 29292843
Dinutuximab/ch14.18	GD2-directed chimeric monoclonal antibody	n=35	Pain, any grade: 59-80%	PMID: 28549783
		n=53	Pain, Grade 3/4:4-51%	PMID: 32343642
		n=25	Motor weakness:4-6%	PMID: 31815885
		n=28	Paresthesias: 6-9%, Encephalopathy: 4-9%	PMID: 26791869
		n=16	Seizure: 1-2%	PMID: 23924804
		n=246	PRES: 1%	PMID: 30442501
		n=53	Toxic demyelinating encephalopathy: 1%	PMID: 29350486
		n=25	Ocular/visual problems: 8-22%	PMID: 19047298
		n=226		PMID: 20879881
		n=11		PMID: 9626218
		n=9		PMID: 7718335
		n=42		PMID: 31601569
Hu14.18K322A	GD2-directed humanized monoclonal antibody	n=13	Pain, any grade: 100%	PMID: 31601569
		n=30	Pain, Grade 3/4:17-68%	PMID: 28939747
		n=38	Encephalopathy: 3%	PMID: 28733263
			Ocular/visual problems: 42-54%	PMID: 24711551
3F8	GD2-directed murine monoclonal antibody	n=34	Pain, any grade: Described as common/100%	PMID: 9738575
		n=16	Seizure 2%	PMID: 9592190
		n=79	PRES 2.3%	PMID: 24644014
		n=19	Ocular/visual problems: 9%	PMID: 11709561
		n=215	Atonia 5%	PMID: 23633099
		n=57		PMID: 30326045
14.G2a	GD2-directed murine monoclonal antibody	n=33	Pain, any grade: 27%-100%	PMID: 9217046
		n=18	Paresthesia: 42-66% Motor weakness: 0-33%	PMID: 8270976
		n=9	Encephalopathy: 0-33%	PMID: 1638557
			Ocular/visual problems: 11%	
Hu14.18-IL2	GD2-directed humanized monoclonal antibody linked to IL-2	n=27	Pain, any grade: 31-64%	PMID: 16551859
		n=51	Headache: 10%	PMID: 31358541
		n=39		PMID: 20921469
131-1-3F8	GD2-directed murine monoclonal antibody radiolabeled with iodine-131	n=43	Antibody delivered intrathecally to patients with high-risk/recurrent medulloblastoma	PMID: 28940863
		n=13	< Grade 2 Headaches were commonly seen, most often after the first dose	PMID: 18048828
		n=24	Rare grade 3 events were seen: headaches, CSF pleocytosis consistent with chemical meningitis, and acute dystonic reaction	PMID: 11464891
			In solid tumors:	
			Headache: 100% (Intra-Omaya delivery)	
			Pain: Most (Systemic delivery)	
**Antibody-drug conjugate**				
Inotuzumab ozogamicin	CD22-directed humanized monoclonal antibody that is conjugated to calicheamicin, is rapidly internalized into the cell, forms an endosome which subsequently fuses with lysosomes and mediates cellular apoptosis.	n=25	Infrequently causes neurotoxicity with headaches seen most commonly (28%), and bone pain reported in a minority of patients. No > grade 3 events noted	PMID: 30267011
				PMID: 33067614
Brentuximab vedotin	CD30-directed antibody linked to a microtubule disrupting agent that selectively induces apoptosis in CD-30 positive cells	n=36	One-third of patients developed peripheral neuropathy. Other notable toxicities included paresthesia, myalgia, headache, and pain	PMID: 30290902
				PMID: 33826362
Lorvotuzumab mertansine	CD56-directed humanized monoclonal antibody that is linked to an antimitotic agent	n=62	Headache (1%)	PMID: 32914879
			Motor neuropathy (1%)	
			Sensory neuropathy (1%)	
Glembatumumab vedotin	Glycoprotein NMB-directed human monoclonal antibody linked to a microtubule inhibitor	n=22	Pain (9%)	PMID: 31586757
			Headache (1%)	
			Somnolence (1%)	
			Sensory neuropathy (1%)	
**Vaccines**				
H3.3 K27M	H3.3 K27M targeted peptide vaccine	n=29	Headaches (38%)	PMID: 32817593
			Gait disturbances (17%)	
			Weakness (13.8%)	
			Cranial nerve palsies	
WT1	WT1 targeted peptide vaccine		No Neurotoxicity reported	PMID: 26469989
				PMID: 32793489
				PMID: 29599343
Glioma-associated antigen vaccine	EphA2, IL13Ra2, surviving targeted peptide vaccine	n=26	Several neurologic AEs have been reported, cranial nerve palsy, central hypoventilation, torticollis, intratumoral hemorrhage, gait disturbances, with difficulty discerning progressive disease from immune related pseudoprogression	PMID: 24888813
				PMID: 26984745
				PMID: 27624914
Dendritic cell vaccines	Peripheral blood monocytes are enriched for dendritic cells (DCs) by culturing with cytokines, and then the DCs are loaded either with specific peptide antigens, or tumor lysate	n=56	Headaches (16%)	PMID: 18483377
			Focal neurologic symptoms (11%)	PMID: 19852061
			Peritumoral edema (1.8%)	PMID: 23645755
			Rare intratumoral hemorrhage	

MOA, mechanism of action; CAR, chimeric antigen receptor; PD, programmed death, PDL, programmed death-ligand; CNS, central nervous system; nAEs, neurologic adverse events; VEGF-A, vascular endothelial growth factor A.

#### Central Nervous System Tumors

With ICIs, two types of neurologic toxicities can be expected: systemic immune-mediated neurologic disorders, which can occur with ICI use for any indication, and local immune activation at the tumor site, which is unique to brain tumors. Although these presentations are typically easy to distinguish, there may be overlap, and both will likely respond to steroids.

No randomized controlled trial results have yet been published on the use of ICI for treatment of pediatric brain tumors. Efficacy against malignant gliomas in pediatric patients with congenital mismatch repair deficiency (CMMRD) has been described in case reports, with clinical trials ongoing. Two siblings with CMMRD and glioblastoma responded to nivolumab, with their courses complicated by focal seizures and reversible radiographic pseudoprogression (123). In children without CMMRD, transient radiographic responses were seen in 3 of 10 patients with a variety of malignant CNS tumors, and no neurologic toxicities were reported (124). After treatment with 3 doses of nivolumab, a 10-year-old with glioblastoma developed successively worsening severe peritumoral edema; the first time it was reversible with steroids, the second time she required hemicraniectomy, and the third time she died (125). In this case, it was difficult to discern whether this death was from immune response or from tumor progression.

### Pathophysiology, Risk Factors, Evaluation, and Management of ICI-Related Neurotoxicity

The mechanisms of checkpoint-blockade induced neurotoxicity are incompletely understood. Since most ICI toxicities have a high degree of overlap with known autoimmune or inflammatory conditions, failure of self-tolerance (1) and contributions from B cell and T cell mediated mechanisms are suspected (126). Indeed, increased clonal T cell expansion is associated with higher risk of ICI toxicity (127). Autoantibodies are frequently found in patients with ICI-related nAEs, with profiles distinct from but overlapping with typical paraneoplastic antibody profiles (128, 129). Thus, patients with preexisting autoimmune neurologic conditions such as multiple sclerosis or myasthenia gravis are at risk of relapse during ICI treatment (115, 116, 130).

Work up for neurotoxicity may include neuro-imaging, CSF studies, ophthalmologic exam, neurophysiologic testing for peripheral nerve pathologies, and antibody titers (131, 132). An endocrine workup should also be considered given the high incidence of endocrine complications during ICI treatment, particularly with ipilimumab (133).

Treatment of neurologic conditions related to ICI treatment is generally similar to interventions for when the same syndromes occur without ICI use. There is no evidence beyond expert opinion to support this approach. Depending on the severity of irAEs, immunotherapy may need to be paused or discontinued. If this is not sufficient, immunomodulators, primarily corticosteroids, may be indicated, as laid out in the Society for Immunotherapy of Cancer (SITC) and American Society of Clinical Oncology (ASCO) consensus guidelines of care for ICIs (11, 12, 134). For nAEs that are proven or suspected to be autoantibody-mediated, such as Guillain-Barré syndrome, IVIG or plasmapheresis are recommended (131).

### Antibody-Based Therapy

#### General

Antibody-based therapy in cancer therapeutics utilizes the recognition properties of an antibody to bind to a specific antigen on a malignant cell. Upon binding, the immune system triggers downstream processes that promote cell destruction and apoptosis either through direct cytotoxicity, non-restricted activation of cytotoxic T cells, and/or Fc-mediated immune effector engagement (135). The toxicity profile of the antibody-based therapy will be dependent on whether an antibody is conjugated (bound to another substrate such as an enzyme, toxin, or inorganic compound) or unconjugated, as conjugation itself can alter the side effect profile. This section will focus on three categories, bispecific T cell engagers, unconjugated antibodies, and antibody-drug conjugates.

### Hematologic Malignancies

#### Bispecific T Cell Engager

##### Blinatumomab

Antibody-based therapy in pediatric hematologic malignancies has demonstrated great clinical success in treating patients with relapsed/refractory acute lymphoblastic leukemia. Blinatumomab is a 55 kD-fusion protein that is a bispecific T cell engager that binds to and activates CD3+ T cells and links these T cells to the CD19 surface antigen on leukemia cells. Blinatumomab creates a synapse between the T cells and CD19+ cells, which results in upregulation of cell adhesion molecules, production of cytolytic proteins, release of inflammatory cytokines, and causes proliferation of T cells which results in lysis of CD19+ cells (136). Several studies have been published demonstrating complete remission rates of 30-50% in patients with relapsed/refractory ALL, with improved disease-free survival as compared to chemotherapy cohorts alone (137–139). Cytokine release syndrome and neurotoxicity are serious and common side effects that can occur with blinatumomab (136).

##### Neurotoxicity Experience in Blinatumomab

In pediatric patients with ALL receiving blinatumomab, neurologic toxicities have occurred in approximately 32% of patients in published reports (137–148). Most events occur early within the first treatment cycle and are usually associated with the start of the infusion or during the dose escalation phase. The most common neurologic manifestations in pediatric ALL patients are headache and tremor. Grade 3 or higher neurologic toxicities following initiation of blinatumomab occur in approximately 5% of pediatric patients and included seizures, encephalopathy, tremor, neuralgia, depressed level of consciousness, dysarthria, agitation, cranial nerve disorder, somnolence, and headaches (137–148). It is important to note that there is extremely limited data (case reports) of patients with active central nervous system ALL receiving blinatumomab as these patients were excluded from clinical trials, thus the incidence of neurotoxicity in this population is unknown.

##### Pathophysiology of Blinatumomab-Related Neurotoxicity

The pathophysiology of neurotoxicity post-blinatumomab is elusive and remains an active area of research. T cell adhesion to blood vessel endothelium, subsequent endothelial activation, and T cell migration into the perivascular space has been described as a potential mechanism for neurotoxicity (149). Additionally, histopathologic examinations and analyses of CSF in patients treated with blinatumomab demonstrated T cells at the brain microvascular endothelium and in the CSF. Interestingly, blinatumomab was detectable in the CSF, including in those patients with no to mild disturbance of their blood-CSF barrier (measured by CSF albumin concentration) (149). However, no direct relationship with CSF levels of blinatumomab and neurotoxicity have been seen.

##### Risk Factors for Blinatumomab Neurotoxicity

Risk factors in pediatric patients for development of neurotoxicity with blinatumomab treatment are not well defined, however higher doses of blinatumomab have correlated with higher incidence of neurotoxicity (150). In adult patients, a lower B:T cell ratio has been demonstrated to increase incidence of nAEs (149), however this has not been validated in pediatric patients.

##### Evaluation and Management of Blinatumomab-Related Neurotoxicity

In patients who develop treatment-associated neurotoxicity, work up should be performed based on symptoms present and severity, with neuroimaging and EEG performed in those with suspected seizures or severe nAEs. The majority of events resolve with a short interruption in the infusion, dose de-escalation, or complete discontinuation of therapy, and corticosteroids can be used in the presence of severe neurotoxicity. Very few fatalities in pediatric and young adult patients have occurred post blinatumomab (148, 149).

#### Monoclonal, Unconjugated, and Conjugated Antibodies

Targeted therapy using monoclonal, conjugated, and unconjugated antibodies has been utilized in early phase clinical trials, with some drugs now included in upfront treatment in hematologic malignancies. These include antibodies targeting CD20 (e.g., rituximab), CD22 (e.g., inotuzumab, epratuzumab), and CD30 (e.g., brentuximab vedotin) with specific clinical trials discussed in **Table 3**. Broadly, use of these antibodies in pediatric hematologic malignancies has shown an incidence of neurotoxicity of up to 30% in published trials, with the most common neurologic adverse events being pain, peripheral neuropathy, and headache (151–154). Other more rare and serious adverse events seen include chorioretinitis, cerebellar syndrome, cognitive disturbances, encephalopathy, intracranial hemorrhage, and meningitis (155, 156).

### Solid Tumors

#### Bispecific T Cell Engager

Clinical trials with BITEs in pediatric solid tumor are ongoing with no toxicity data available.

#### Monoclonal Antibodies

Many unmodified and modified monoclonal antibodies have been evaluated for high-risk, relapsed, and refractory solid tumors. Reported neurotoxicity from published clinical trials on specific agents is summarized in **Table 3**. In clinical trials evaluating multiple agents, it is not possible to determine if neurotoxicity is directly caused by the monoclonal antibody. The disialoganglioside GD2 is a common monoclonal antibody target in pediatric oncology. Neuropathic pain is a well-described toxicity of anti-GD2 antibody therapy due to on-target binding of pain fibers and activation of the complement system (157). Co-administration of nonsteroidal anti-inflammatory drugs, gabapentinoids, and analgesic drugs, as well as temporarily stopping and slowing the infusion are primary ways to mitigate pain in patients. Common sites of pain are the abdomen, chest, back, and extremities. Pain is usually observed shortly after the start of the infusion, is most frequent during the first cycle of anti-GD2 therapy, and typically decreases in frequency during subsequent cycles (158).

Adie’s pupil is an ocular abnormality associated with anti-GD2 antibody therapy. It is characterized by mydriasis and accommodation deficits and is thought to be due to binding of antibody to neural structures in the ciliary and sphincter muscles of the eye. Most vision abnormalities can be corrected with prescription glasses and symptoms often improve or completely resolve after completion of therapy (159).

Posterior reversible encephalopathy syndrome (PRES) is also associated with GD2 targeting antibody therapy and can present with hypertension, seizures, headache, visual disturbance, and/or altered mentation. Characteristic radiographic findings of PRES on MRI of the brain are edematous changes most often seen in the parietal and occipital lobes. Prior brain irradiation is significantly associated with incidence of PRES in patients who received the anti-GD2 antibody, 3F8. Most patients have complete resolution of symptoms over weeks (160).

### CNS Tumors

#### Bispecific T-Cell Engagers

Bispecific T cell engagers are in clinical trials for pediatric CNS tumors however toxicity data is not yet available.

#### Monoclonal, Unconjugated, and Conjugated Antibodies

Bevacizumab is the most frequently used antibody-drug in brain tumors (161, 162). It acts by blocking angiogenesis *via* neutralizing free circulating VEGF-A. Since it reduces contrast enhancement of tumors, interpretation of tumor response by imaging criteria alone is problematic. In pediatric trials, bevacizumab has been used together with chemotherapy and/or radiation. While safety profiles are favorable, efficacy has not been well established due to small trial size. Serious nAEs are rare; the most common of these are intracranial hemorrhages (ICH), which affect 1-4% of patients and are typically intratumoral (163–166). Importantly however, control groups not receiving bevacizumab have similar rates of ICH, which has been seen in primary brain tumors in children and adults, as well as in those with brain metastases (167–170). Other rare but serious reported adverse events include CNS ischemia (**Table 3**) (165).

EGFR is frequently expressed by pediatric brain tumors making EGFR a suitable epitope for tumor-targeting immunotherapies (171). The antibody drugs cetuximab, nimotuzumab, and depatuxizumab (172) have been tested in children, and generally have been well tolerated (173, 174), with rare cases of headaches and ICH seen. Depatuxizumab, which targets a tumor specific EGFR epitope, has been shown to be safe in adult studies (175). However, the drug-antibody conjugate depatuxizumab mafodotin has caused corneal keratopathy in 33-81% of adult patients with GBM (175, 176). Pediatric trials of depatuxizumab mafodotin for HGG have been completed but not yet been published.

Multiple radiolabeled antibody conjugates are in clinical trials for pediatric brain tumors but no toxicity data is available yet.

## Vaccine Based Therapy

### General

Vaccines for oncologic therapeutics contain tumor-associated antigens that work by engaging the immune system to recognize and react to these antigens and destroy cancer cells that contain them. The goal of therapeutic cancer vaccines is to induce tumor regression, eradicate minimal residual disease, and establish lasting anti-tumor memory (177). Neurologic toxicities have only been described in vaccine trials for brain tumors, where distinction between tumor progression and irAE is extremely challenging. Although it is plausible that a tumor-directed immune response could cause peritumoral inflammation, this has not been conclusively demonstrated. Thus, there is currently no evidence for a specifically immune-related neurologic syndrome associated with tumor vaccine.

### Hematologic Malignancies

In pediatric hematologic malignancies, there is limited experience utilizing vaccines for therapeutic interventions. Wilms tumor gene, *WT1*, vaccine has been used with limited clinical data in patients with acute leukemias, either in the setting of minimal residual disease post hematopoietic stem cell transplant or in those with relapsed disease, without any reported neurotoxicity (178, 179).

### Solid Tumors

There are a limited number of published clinical trials utilizing tumor antigen-derived peptide and dendritic cell vaccines in pediatric solid tumors. One study evaluating the NCCV Cocktail-1 vaccine, a combination of peptides derived from the KOC1, FOXM1, and KIF20A cancer antigens, described mild headache as a toxicity (16.7%) (180). Some vaccine studies reported injection site pain (180–183). No neurotoxicity, including complaints of pain, were noted in all other published vaccine trials for pediatric patients with solid tumors (184–186). All published vaccine studies in pediatric patients thus far have shown safety, feasibility, and some anti-tumor activity.

### CNS Tumors

Tumor vaccines have been the most extensively studied immunotherapy in the pediatric brain tumor population (187). Peptide vaccines to sensitize against tumor-specific antigens have been safe, but few antitumor responses have been described despite successful sensitization. Injection-site reactions and mild flu-like symptoms including fever and headache occur in most patients. When nAEs occurred, they were focal in nature (e.g., gait disturbances, weakness, cranial nerve palsies) and attributable to the primary tumor site, with a differential diagnosis of tumor progression versus immune-related pseudoprogression (188–190). (See **Table 3** for more vaccine and trial specific details).

## Summary

Pediatric cancer immunotherapy comprises a diverse set of approaches for heterogeneous diseases, with distinct biology and mechanism of action of immunotherapy approaches. This makes it inherently challenging to draw conclusions on risks and benefits given small sample sizes. Current efforts are focused primarily on creation of novel immunotherapies to target tumors more effectively, and limit broad, more systemic toxicities seen with chemotherapy and radiation therapy. However, the emergence of neurotoxicity as a key adverse event in immune cell engaging therapies for hematologic malignancies has invigorated interest in this topic in the pediatric population.

Compared with the ability to create novel targeted therapies and our level of understanding on efficacy and target choice of immunotherapies, the mechanistic understanding of these novel toxicity profiles is lagging, at least in part due to lack of suitable *in vitro* and *in vivo* models to help evaluate these toxicities. Although the mechanisms of neurotoxicity remain unsolved, efforts at understanding the pathophysiology of this toxicity must be boosted to engineer safer novel therapies and inform diagnostic and therapeutic toxicity guidelines. For instance, based on pre-clinical models noting increased IL-1 in xenograft models post-CAR therapy, several clinical trials are now investigating use of anakinra as a therapeutic strategy to mitigate neurotoxicity. Future directions should focus on collaborative larger scale trials to enhance data sharing and to develop algorithms for early detection and effective treatment strategies for neurotoxicity.

Additionally, through multicenter trials, prospective evaluation of biomarkers and ancillary studies such as long-term neurocognitive assessments can be performed and may help validate biomarkers and enhance clinicians ability to identify patients at risk of developing acute and long-term neurotoxicity after novel immunotherapies.

## Author Contributions

All authors planned and wrote the manuscript and contributed to the article and approved of the submitted version.

## Funding

This work was supported in part by the Intramural Research Program, Center of Cancer Research, National Cancer Institute and NIH Clinical Center, National Institutes of Health (ZIA BC 011823, N.N.S), and the National Institute of Neurological Disorders and Stroke Mentored Clinical Scientist Research Career Development Award (1K08NS118138-01, JG).

## Author Disclaimer

The content of this publication does not necessarily reflect the views of policies of the Department of Health and Human Services, nor does mention of trade names, commercial products, or organizations imply endorsement by the U.S. Government.

## Conflict of Interest

The authors declare that the research was conducted in the absence of any commercial or financial relationships that could be construed as a potential conflict of interest.

## Publisher’s Note

All claims expressed in this article are solely those of the authors and do not necessarily represent those of their affiliated organizations, or those of the publisher, the editors and the reviewers. Any product that may be evaluated in this article, or claim that may be made by its manufacturer, is not guaranteed or endorsed by the publisher.
